# State-Level Variability in Hospital Presumptive Eligibility Programs

**DOI:** 10.1001/jamanetworkopen.2023.45244

**Published:** 2023-11-28

**Authors:** Alexander B. Gibson, Wesley D. Hendricks, Katherine Arnow, Linda D. Tran, Todd H. Wagner, Lisa Marie Knowlton

**Affiliations:** 1 Stanford-Surgery Policy Improvement Research and Education Center (S-SPIRE), Stanford, California; 2 Section of Trauma, Surgical Critical Care and Acute Care Surgery, Stanford University School of Medicine, Stanford, California

## Abstract

This cross-sectional study examines state-level variability in hospital presumptive eligibility programs to understand discrepancies in access by Medicaid expansion status.

## Introduction

The Patient Protection and Affordable Care Act (ACA) mandated hospital presumptive eligibility (HPE), a temporary form of Medicaid coverage granted to uninsured patients in an emergency. HPE offers comprehensive Medicaid services beginning the day of HPE approval for up to 60 days, but patients must file a full Medicaid application to continue coverage. Eligibility varies by state, including but not limited to patients with incomes at or below the 133% federal poverty level, pregnant women and children, patients with cancer, or patients with disability. In this cross-sectional study, we examined state-level variability in HPE programs to understand discrepancies in access by Medicaid expansion status.

## Methods

We conducted a systematic web-based search between June 1 and June 30, 2023, on official state websites to assess current 2023 HPE eligibility in all 50 states and Washington, DC. We identified 8 coverage groups: pregnant women, children aged 0 to 19 years, former foster children, parents or caregivers, adults aged 19 to 64 years, patients with breast or cervical cancer, individuals needing family planning services, and adults aged 65 years and older (eAppendix in Supplement 1). Eligibility was compared by state Medicaid expansion status to understand expected and unanticipated differences in HPE coverage. We further qualified this by tallying the eligible groups covered by each state, assigning a generosity score (0 to 8, with higher scores indicating a greater number of covered groups).^1^ We investigated states’ residency and immigration verification and accessibility of online HPE information. Our study was exempt from institutional review board approval and the need for informed consent because no individual patient data were used, in accordance with 45 CFR §46, and follows STROBE reporting guidelines. Data were managed with Excel software version 16.78.3 (Microsoft).

## Results

We analyzed HPE eligibility across 50 states and Washington, DC, including 41 Medicaid-expanded states (80.3%) and 10 nonexpanded states (19.7%). Compared with Medicaid-expanded states, nonexpanded states had higher rates of HPE coverage for former foster children (100.0% vs 78.0%), parents or caregivers (100.0% vs 75.6%), and patients with breast or cervical malignant neoplasm (60.0% vs 51.2%) (Table). Conversely, 75.6% of Medicaid expanded sites covered all adults aged 19 to 64 years, compared with 10.0% of nonexpanded states.

**Table.** US States Meeting Certain Hospital Presumptive Eligibility Criteria (Medicaid Expanded vs Nonexpanded)

Eligibility criteria	States, No. (%)		
	All states and Washington, DC (N = 51)	Expanded states (n = 41)^a^	Nonexpanded states (n = 10)
Pregnant women	51 (100.0)	41 (100.0)	10 (100.0)
Children (aged 0-19 y)	51 (100.0)	41 (100.0)	10 (100.0)
Former foster children	42 (82.4)	32 (78.0)	10 (100.0)
Parent or caregiver	41 (80.4)	31 (75.6)	10 (100.0)
Adults (aged 19-64 y)	32 (62.7)	31 (75.6)	1 (10.0)
Breast and cervical cancer group	27 (52.9)	21 (51.2)	6 (60.0)
Family planning group	15 (29.4)	13 (31.7)	2 (20.0)
Age ≥65 y	2 (3.9)	2 (4.9)	0

^a^ Values were determined including North Carolina, which has proposed expansion within the year.

There was substantial variability in HPE generosity (Figure). Six states covered 7 groups. Three states only covered 2 groups. We found that 90.0% of states required state residency and US citizenship; 86.0% of states allowed for patient attestation of residency and citizenship status. Regarding accessibility, 17 states presented HPE information on their government website; 7 lacked required supplementation with external documents.

**Figure.**
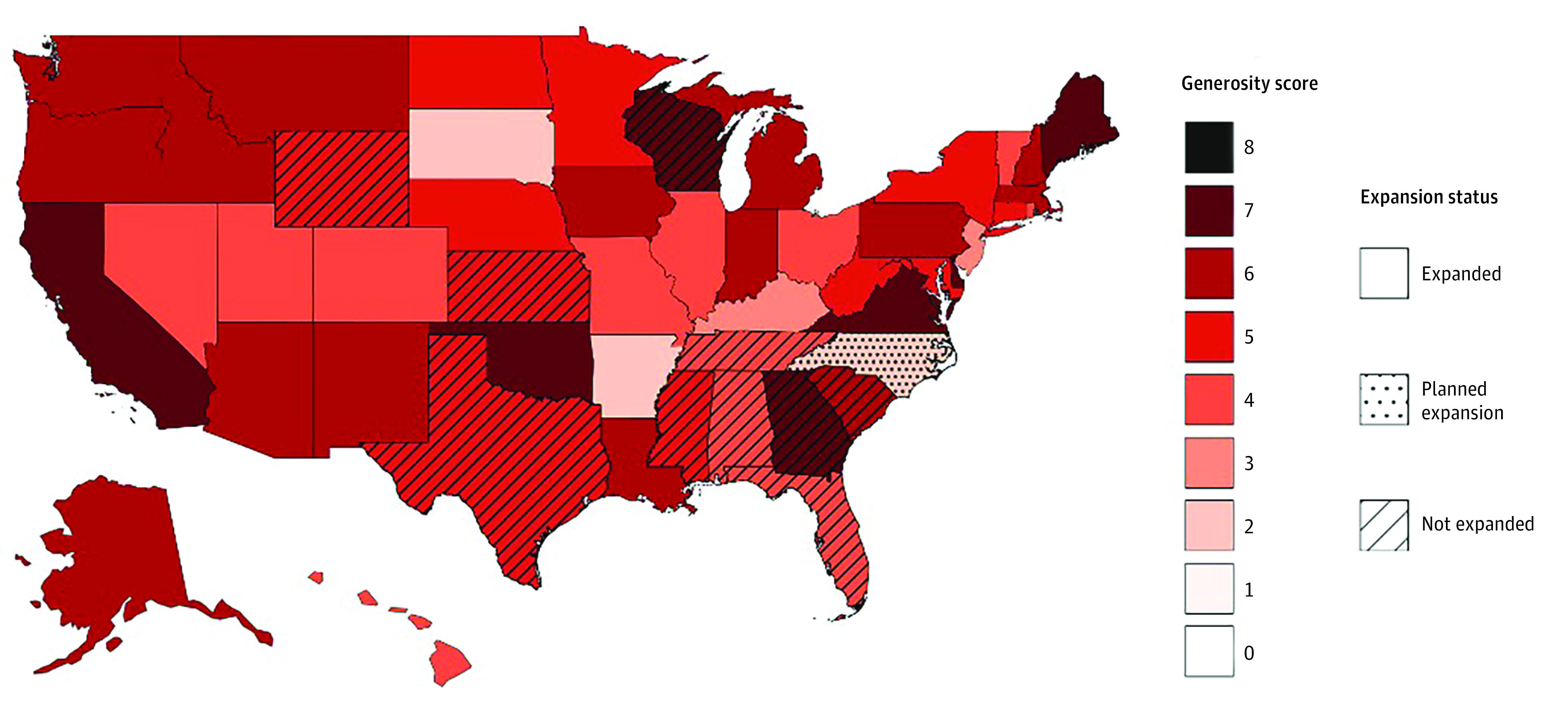
State Variability in Hospital Presumptive Eligibility by Generosity Score The generosity score has a range of 0 to 8, with higher scores indicating a greater number of covered groups. Python software version 3.11.3 (Python Software Foundation) was used to make the map.

## Discussion

HPE, mandated as part of the ACA, provides temporary emergency Medicaid coverage for uninsured patients.^2^ This cross-sectional study found that considerable state-level variation in HPE coverage groups exists. Among nonexpanded states in particular, opportunities exist to extend HPE coverage to all adults aged 19 to 64 years rather than limiting coverage to very focused patient populations.^3^

Policymakers can use HPE programs to provide uninsured patients with short-term necessary medical resources and improved care.^2,4^ Our prior work^2^ has shown that HPE is a pathway to obtaining full scope Medicaid, with 70% of HPE enrollees sustaining Medicaid at 6 months and having increased access to posthospitalization health care services. The expansion of HPE to more coverage groups could allow states to expand access to health care.^5,6^

States can also increase transparency of HPE information for hospitals and patients. In 7 states, availability of HPE information was so limited that patients would encounter challenges learning about HPE from official government websites. This represents an opportunity to improve online educational resources for patients and practitioners. We acknowledge that our findings may have been limited by the accuracy and currency of our internet sources.

Although HPE has been implemented across states, coverage discrepancies persist. Policymakers can further leverage HPE programs to cover more uninsured groups. In addition, states can improve transparency and availability of HPE information for patients and hospitals. Increased accessibility would bolster the ongoing discussion surrounding HPE and its impact on health care access.

## Notes

Supplement 1. **eAppendix.** Background on Patient Groups Covered by HPE
eReferences

Supplement 2.
Data Sharing Statement
