# Supplementary material for: State-Level Variability in Hospital Presumptive Eligibility Programs
Source: JAMA Netw Open. 2023 Nov 28;6(11):e2345244. doi: 10.1001/jamanetworkopen.2023.45244 (PMC10685880; doi:10.1001/jamanetworkopen.2023.45244)
Supplement: Supplement 1. — eAppendix. Background on Patient Groups Covered by HPE eReferences [file jamanetwopen-e2345244-s001.pdf]

Supplemental Online Content

Gibson AB, Hendricks WD, Arnow K, Tran LD, Wagner TH, Knowlton LM. State-level variability in hospital presumptive eligibility programs. *JAMA Netw Open*. 2023;6(11):e2345244. doi:10.1001/jamanetworkopen.2023.45244

eAppendix. Background on Patient Groups Covered by HPE
eReferences

This supplemental material has been provided by the authors to give readers additional information about their work.

© 2023 Gibson AB et al. *JAMA Network Open.*

eAppendix: Background on Patient Groups Covered by HPE

1. Pregnant Women:
Title XIX of the Social Security Act requires states to provide Medicaid coverage to pregnant women with incomes up to a certain threshold.[1]

2. Children (0-19):
The inclusion of children aged 0-19 aligns with the Children's Health Insurance Program (CHIP) and the Early and Periodic Screening, Diagnostic, and Treatment (EPSDT) benefit within Medicaid.[1]

3. Former Foster Children:
The extension of presumptive eligibility to former foster children is supported by the Foster Care Independence Act of 1999.[2]

4. Parent or Caregiver:
Presumptive eligibility for parents or caregivers aligns with the federal requirements set forth in the Affordable Care Act (ACA) of 2010.[3]

5. Adults (19-64):
The inclusion of adults aged 19-64 is consistent with the broader Medicaid expansion efforts under the ACA.[4]

6. Breast and Cervical Cancer Group
The Breast and Cervical Cancer Prevention and Treatment Act of 2000 specifically extended presumptive eligibility to individuals diagnosed with breast or cervical cancer.[5]

7. Family Planning Group
The Title X Family Planning Program ensures access to comprehensive family planning services, including contraception and related preventive health services.[6]

8. 65+
Individuals aged 65 and older are eligible for Medicare, a federally administered program. However, individuals in this group may be dually eligible for Medicaid and Medicare services in some states.[7]

eReferences

1. SSA O. Medicaid and CHIP Payment and Access Commission. Accessed October 8, 2023. https://www.ssa.gov/OP_Home/ssact/title19/1900.htm

2. OLCA. The Foster Care Independence Act of 1999. Accessed October 8, 2023. https://www.ssa.gov/legislation/legis_bulletin_112499.html

3. The Affordable Care Act and Caregivers Research Brief. ASPE. Accessed October 8, 2023. https://aspe.hhs.gov/reports/affordable-care-act-caregivers-research-brief-0

4. PLAW-111publ148.pdf. Accessed October 8, 2023. https://www.govinfo.gov/content/pkg/PLAW-111publ148/pdf/PLAW-111publ148.pdf

5. 2000 - Public Law 106-354 106th Congress.pdf. Accessed October 8, 2023. https://ftp.cdc.gov/pub/publications/cancer/nbccedp/pdf/publ354-106.pdf

6. IF10051.pdf. Accessed October 8, 2023. https://crsreports.congress.gov/product/pdf/IF/IF10051

7. Medicare and Medicaid Act (1965). National Archives. Published October 5, 2021. Accessed October 8, 2023. https://www.archives.gov/milestone-documents/medicare-and-medicaid-act

© 2023 Gibson AB et al. *JAMA Network Open.*
