# Supplementary material for: State-Level Variability in Hospital Presumptive Eligibility Programs
Source: JAMA Netw Open. 2023 Nov 28;6(11):e2345244. doi: 10.1001/jamanetworkopen.2023.45244 (PMC10685880; doi:10.1001/jamanetworkopen.2023.45244)
Supplement: Supplement 2. — Data Sharing Statement [file jamanetwopen-e2345244-s002.pdf]

Data Sharing Statement

Gibson. State-Level Variability in Hospital Presumptive Eligibility Programs. *JAMA Netw Open*. Published November 28, 2023. doi:10.1001/jamanetworkopen.2023.45244

Data
Data available: No

Additional Information
Explanation for why data not available: publicly available data
